# Supplementary material for: A 7T fMRI dataset of synthetic images for out-of-distribution modeling of vision
Source: Nat Commun. 2026 Feb 9;17:1589. doi: 10.1038/s41467-026-69345-9 (PMC12901131; doi:10.1038/s41467-026-69345-9)
Supplement: Supplementary file 1 — Supplementary Information [file 41467_2026_69345_MOESM1_ESM.pdf]

Supplementary information

A 7T fMRI dataset of synthetic images for out-of-distribution modeling of vision

Alessandro T. Gifford[1,2,3,*], Radoslaw M. Cichy[1,2,3,4], Thomas Naselaris[5,6], Kendrick Kay[5,*]

[1] Institute of Psychology, Freie Universität Berlin, Berlin, Germany
[2] Einstein Center for Neurosciences Berlin, Charité – Universitätsmedizin Berlin, Berlin, Germany
[3] Bernstein Center for Computational Neuroscience, Humboldt-Universität zu Berlin, Berlin, Germany
[4] Berlin School of Mind and Brain, Humboldt-Universität zu Berlin, Berlin, Germany
[5] Center for Magnetic Resonance Research, Department of Radiology, University of Minnesota, Minneapolis, MN, USA
[6] Department of Neuroscience, University of Minnesota, Minneapolis, MN, USA

* Correspondence: alessandro.gifford@gmail.com, kay@umn.edu

Supplementary Fig. 1 | NSD-synthetic and NSD-core's noise ceiling signal-to-noise ratio (NCSNR).. 3
Supplementary Fig. 2 | In-distribution and out-of-distribution generalization comparison of AlexNet-based encoding models, controlling for session-specific effects............................... 4
Supplementary Fig. 3 | In-distribution and out-of-distribution generalization comparison for encoding models based on individual AlexNet layers, trained with linear regression............. 5
Supplementary Fig. 4 | In-distribution and out-of-distribution generalization comparison for encoding models based on individual AlexNet layers, trained with ridge regression............... 6
Supplementary Fig. 5 | In-distribution and out-of-distribution generalization comparison of vision-transformer-based encoding models...7
Supplementary Fig. 6 | In-distribution and out-of-distribution generalization comparison of ResNet50-based encoding models.. 8
Supplementary Fig. 7 | In-distribution and out-of-distribution generalization comparison of MoCo-based encoding models..9
Supplementary Fig. 8 | In-distribution and out-of-distribution generalization differences between encoding models, controlling for session-specific effects...................................... 10
Supplementary Fig. 9 | In-distribution generalization differences between encoding models based on individual MoCo and ResNet50 layers, trained with linear regression....................11
Supplementary Fig. 10 | Out-of-distribution generalization differences between encoding models based on individual MoCo and ResNet50 layers, trained with linear regression....... 12
Supplementary Fig. 11 | In-distribution generalization differences between encoding models based on individual MoCo and ResNet50 layers, trained with ridge regression................... 13
Supplementary Fig. 12 | Out-of-distribution generalization differences between encoding models based on individual MoCo and ResNet50 layers, trained with ridge regression........14
Supplementary Fig. 13 | Relationship between the degree of OOD and the generalization performance of AlexNet-based encoding models...15
Supplementary Fig. 14 | Relationship between the degree of OOD and the generalization performance of ResNet50-based encoding models... 17
Supplementary Fig. 15 | Relationship between the degree of OOD and the generalization performance of MoCo-based encoding models..19
Supplementary Fig. 16 | Generalization performance of AlexNet-based encoding models across both naturalistic and synthetic stimulus images...21
Supplementary Fig. 17 | Generalization performance of ResNet50-based encoding models across both naturalistic and synthetic stimulus images...22
Supplementary Fig. 18 | Generalization performance of MoCo-based encoding models across both naturalistic and synthetic stimulus images...23
Supplementary Fig. 19 | Generalization performance of encoding models to naturalistic in-distribution and out-of-distribution test splits chosen based on the image features of a deep neural network..24

Supplementary Fig. 1 | NSD-synthetic and NSD-core's noise ceiling signal-to-noise ratio (NCSNR)

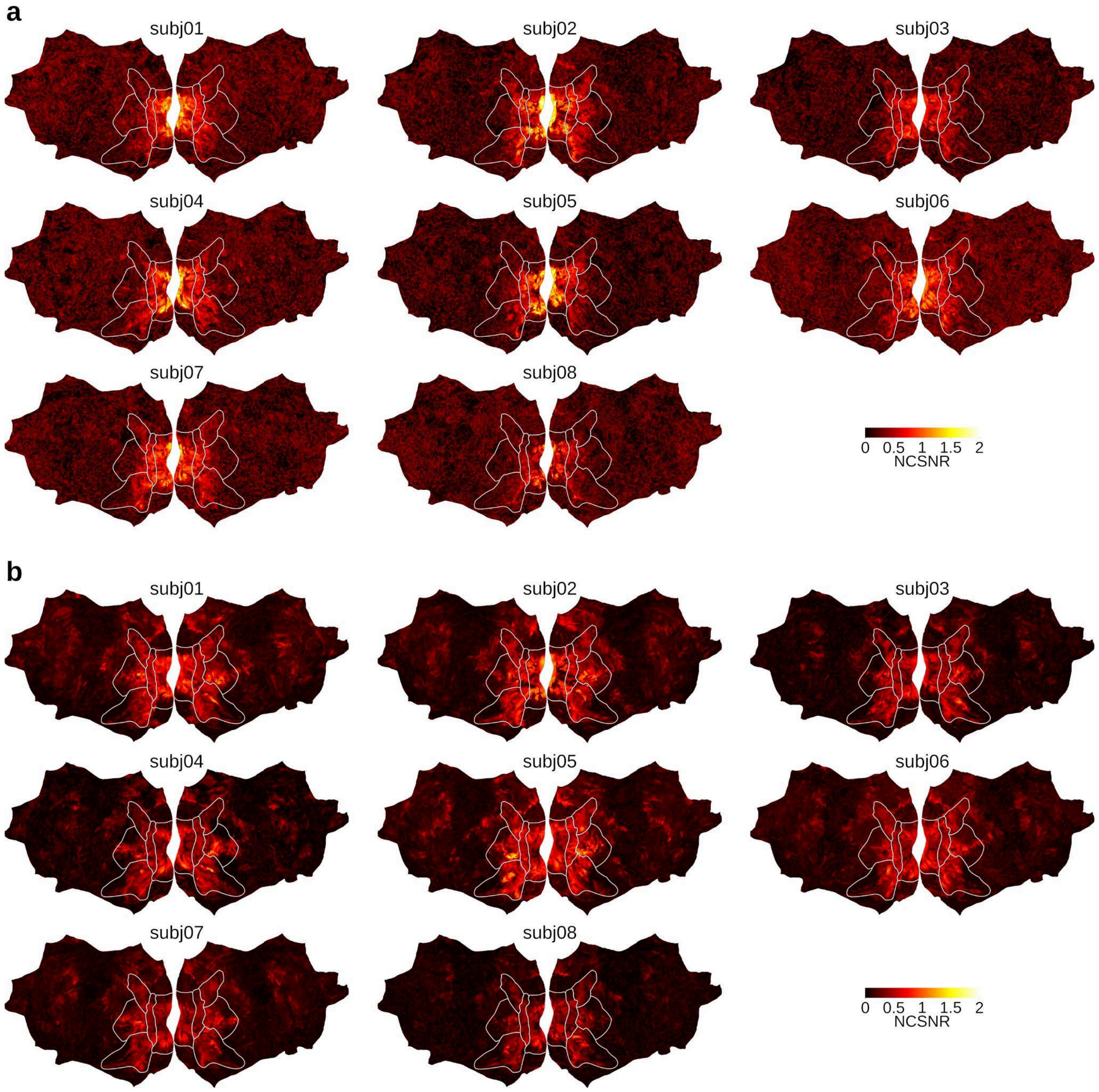

a, NSD-synthetic's participant-wise noise ceiling signal-to-noise ratio (NCSNR), plotted on flattened cortical surfaces. **b,** NSD-core's participant-wise NCSNR, plotted on flattened cortical surfaces. **a-b,** White contours indicate regions based on the 'streams' ROI collection as provided in the NSD data release.

Supplementary Fig. 2 | In-distribution and out-of-distribution generalization comparison of AlexNet-based encoding models, controlling for session-specific effects

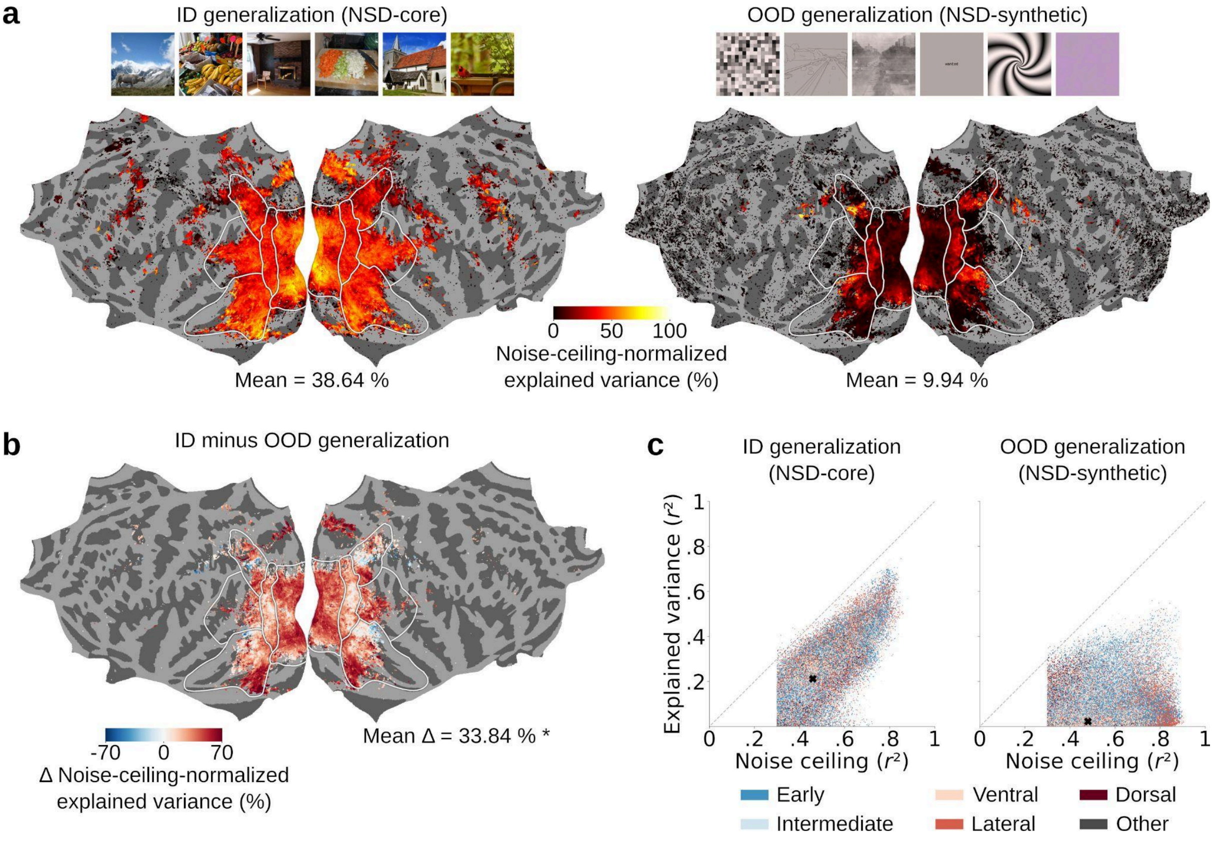

To control for session-specific effects, we trained and tested AlexNet-based encoding models using fMRI responses from separate NSD-core scan sessions. For each participant, we trained the encoding models using image features and fMRI responses from all but the last 5 NSD-core scan sessions. We then tested these models' in distribution (ID) generalization on 284 image conditions that were only presented during NSD-core's last 5 scan sessions, and their out-of-distribution (OOD) generalization on the 284 image conditions from NSD-synthetic. This figure shows results averaged across participants on flattened cortical surfaces. **a,** Model explained variance normalized by the noise ceiling. To reduce false positives, for each vertex we averaged results from only those participants with a noise ceiling greater than 0.3 (vertices for which no participant has a noise ceiling above 0.3 are not shown). **b,** Difference between ID and OOD noise-ceiling-normalized explained variance. For each vertex, we averaged results from only those participants with noise ceiling greater than 0.3 for both NSD-core and NSD-synthetic. The asterisk indicates a significant difference between the ID and OOD noise-ceiling-normalized explained variance scores ($P = 10^{-7}$, paired sample *t*-test, one-sided, $N = 8$ participants). **c,** Scatterplots of vertex-wise explained variance scores against their noise ceilings for ID (NSD-core) and OOD (NSD-synthetic) generalization tests, color coded by the visual stream that each vertex belongs to. For each vertex, we plotted results from only those participants with noise ceiling greater than 0.3 for both NSD-core and NSD-synthetic (same as in panel **b**). Black crosses indicate median scores across all vertices.

Supplementary Fig. 3 | In-distribution and out-of-distribution generalization comparison for encoding models based on individual AlexNet layers, trained with linear regression

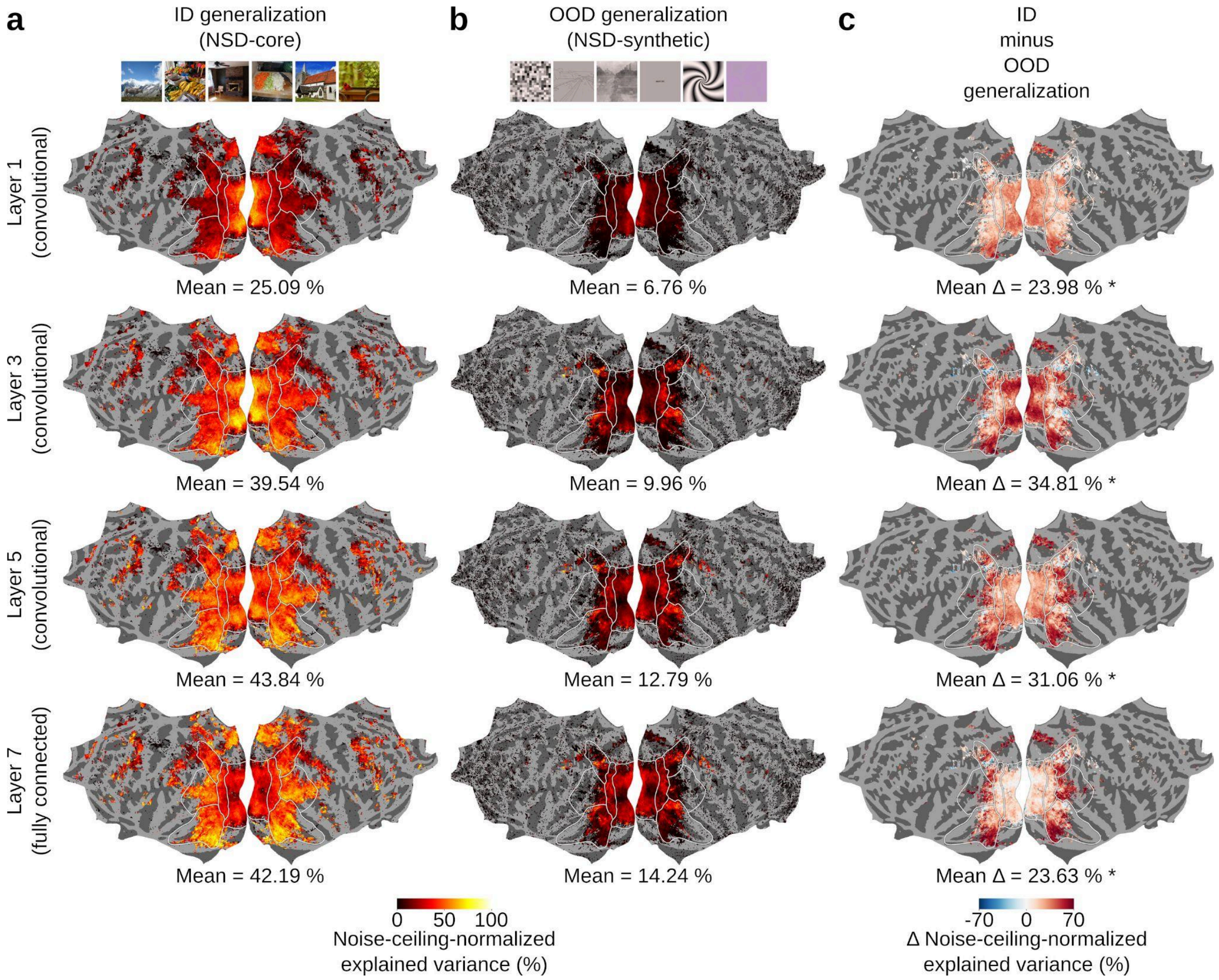

We trained separate encoding models for each of 4 AlexNet layers: convolutional layer 1, convolutional layer 3, convolutional layer 5, and fully connected layer 7. The encoding models consisted of linear regressions that map each layer's image features (downsampled to 250 principal components using PCA) onto the fMRI responses of each vertex. We tested these models in-distribution (ID) on NSD-core as well as out-of-distribution (OOD) on NSD-synthetic. This figure shows results averaged across participants on flattened cortical surfaces. **a,** ID explained variance normalized by the noise ceiling. To reduce false positives, for each vertex we averaged results from only those participants with a noise ceiling greater than 0.3 for NSD-core (vertices for which no participant has a noise ceiling above 0.3 are not shown). **b,** OOD explained variance normalized by the noise ceiling. For each vertex we averaged results from only those participants with a noise ceiling greater than 0.3 for NSD-synthetic. **c,** Difference between ID and OOD noise-ceiling-normalized explained variance. For each vertex, we averaged results from only those participants with noise ceiling greater than 0.3 for both NSD-core and NSD-synthetic. The asterisk indicates a significant difference between the ID and OOD noise-ceiling-normalized explained variance scores ($P < 10^{-6}$, paired sample *t*-test, one-sided, $N = 8$ participants).

Supplementary Fig. 4 | In-distribution and out-of-distribution generalization comparison for encoding models based on individual AlexNet layers, trained with ridge regression

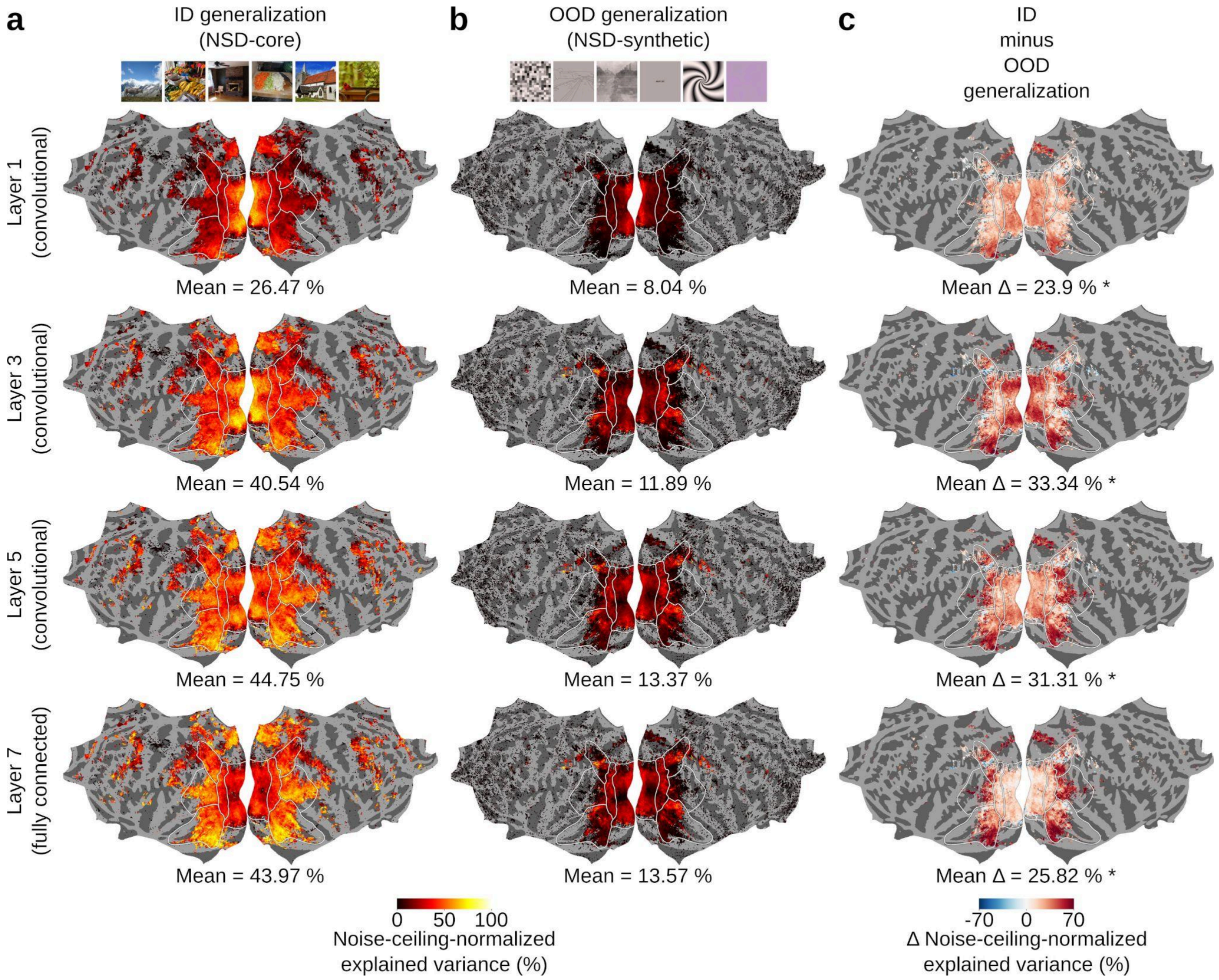

We trained separate encoding models for each of 4 AlexNet layers: convolutional layer 1, convolutional layer 3, convolutional layer 5, and fully connected layer 7. The encoding models consisted of cross-validated ridge regressions that map each layer's image features onto the fMRI responses of each vertex. We tested these models in-distribution (ID) on NSD-core as well as out-of-distribution (OOD) on NSD-synthetic. This figure shows results averaged across participants on flattened cortical surfaces. **a,** ID explained variance normalized by the noise ceiling. To reduce false positives, for each vertex we averaged results from only those participants with a noise ceiling greater than 0.3 for NSD-core (vertices for which no participant has a noise ceiling above 0.3 are not shown). **b,** OOD explained variance normalized by the noise ceiling. For each vertex we averaged results from only those participants with a noise ceiling greater than 0.3 for NSD-synthetic. **c,** Difference between ID and OOD noise-ceiling-normalized explained variance. For each vertex, we averaged results from only those participants with noise ceiling greater than 0.3 for both NSD-core and NSD-synthetic. The asterisk indicates a significant difference between the ID and OOD noise-ceiling-normalized explained variance scores ($P < 10^{-6}$, paired sample *t*-test, one-sided, $N = 8$ participants).

Supplementary Fig. 5 | In-distribution and out-of-distribution generalization comparison of vision-transformer-based encoding models

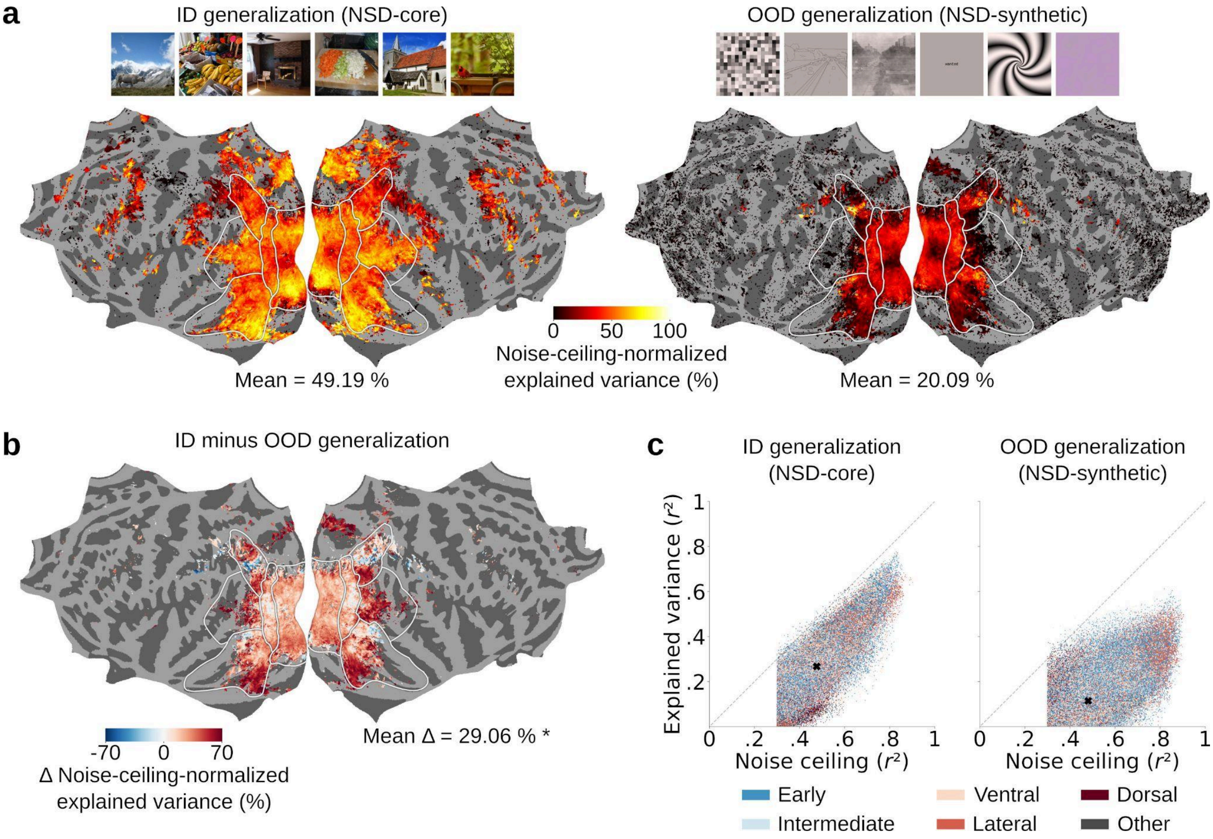

We built vision-transformer-based encoding models and tested them in-distribution (ID) on NSD-core as well as out-of-distribution (OOD) on NSD-synthetic. This figure shows results averaged across participants on flattened cortical surfaces. **a,** Model explained variance normalized by the noise ceiling. To reduce false positives, for each vertex we averaged results from only those participants with a noise ceiling greater than 0.3 (vertices for which no participant has a noise ceiling above 0.3 are not shown). **b,** Difference between ID and OOD noise-ceiling-normalized explained variance. For each vertex, we averaged results from only those participants with noise ceiling greater than 0.3 for both NSD-core and NSD-synthetic. The asterisk indicates a significant difference between the ID and OOD noise-ceiling-normalized explained variance scores ($P = 10^{-7}$, paired sample *t*-test, one-sided, $N = 8$ participants). **c,** Scatterplots of vertex-wise explained variance scores against their noise ceilings for ID (NSD-core) and OOD (NSD-synthetic) generalization tests, color coded by the visual stream that each vertex belongs to. For each vertex, we plotted results from only those participants with noise ceiling greater than 0.3 for both NSD-core and NSD-synthetic (same as in panel **b**). Black crosses indicate median scores across all vertices.

Supplementary Fig. 6 | In-distribution and out-of-distribution generalization comparison of ResNet50-based encoding models

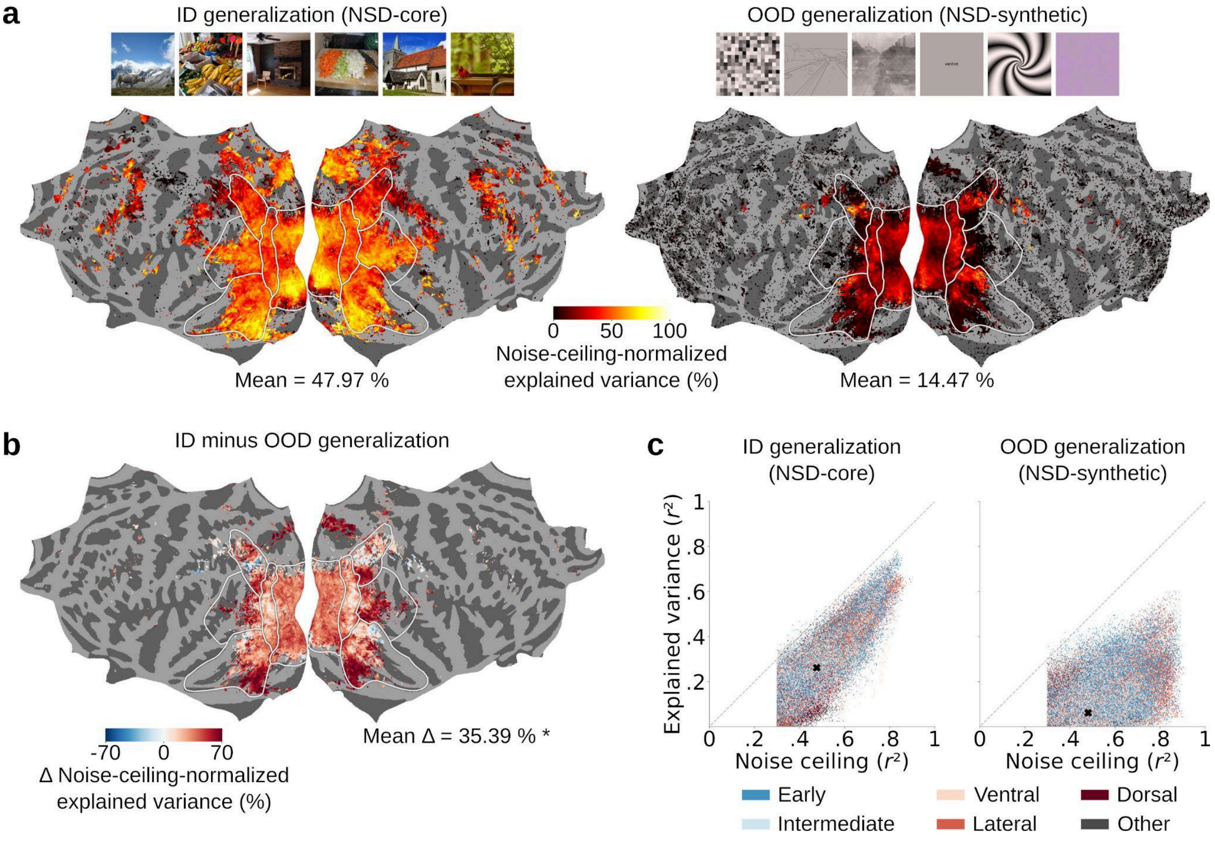

We built ResNet50-based encoding models and tested them in-distribution (ID) on NSD-core as well as out-of-distribution (OOD) on NSD-synthetic. This figure shows results averaged across participants on flattened cortical surfaces. **a,** Model explained variance normalized by the noise ceiling. To reduce false positives, for each vertex we averaged results from only those participants with a noise ceiling greater than 0.3 (vertices for which no participant has a noise ceiling above 0.3 are not shown). **b,** Difference between ID and OOD noise-ceiling-normalized explained variance. For each vertex, we averaged results from only those participants with noise ceiling greater than 0.3 for both NSD-core and NSD-synthetic. The asterisk indicates a significant difference between the ID and OOD noise-ceiling-normalized explained variance scores ($P = 10^{-8}$, paired sample *t*-test, one-sided, $N = 8$ participants). **c,** Scatterplots of vertex-wise explained variance scores against their noise ceilings for ID (NSD-core) and OOD (NSD-synthetic) generalization tests, color coded by the visual stream that each vertex belongs to. For each vertex, we plotted results from only those participants with noise ceiling greater than 0.3 for both NSD-core and NSD-synthetic (same as in panel **b**). Black crosses indicate median scores across all vertices.

Supplementary Fig. 7 | In-distribution and out-of-distribution generalization comparison of MoCo-based encoding models

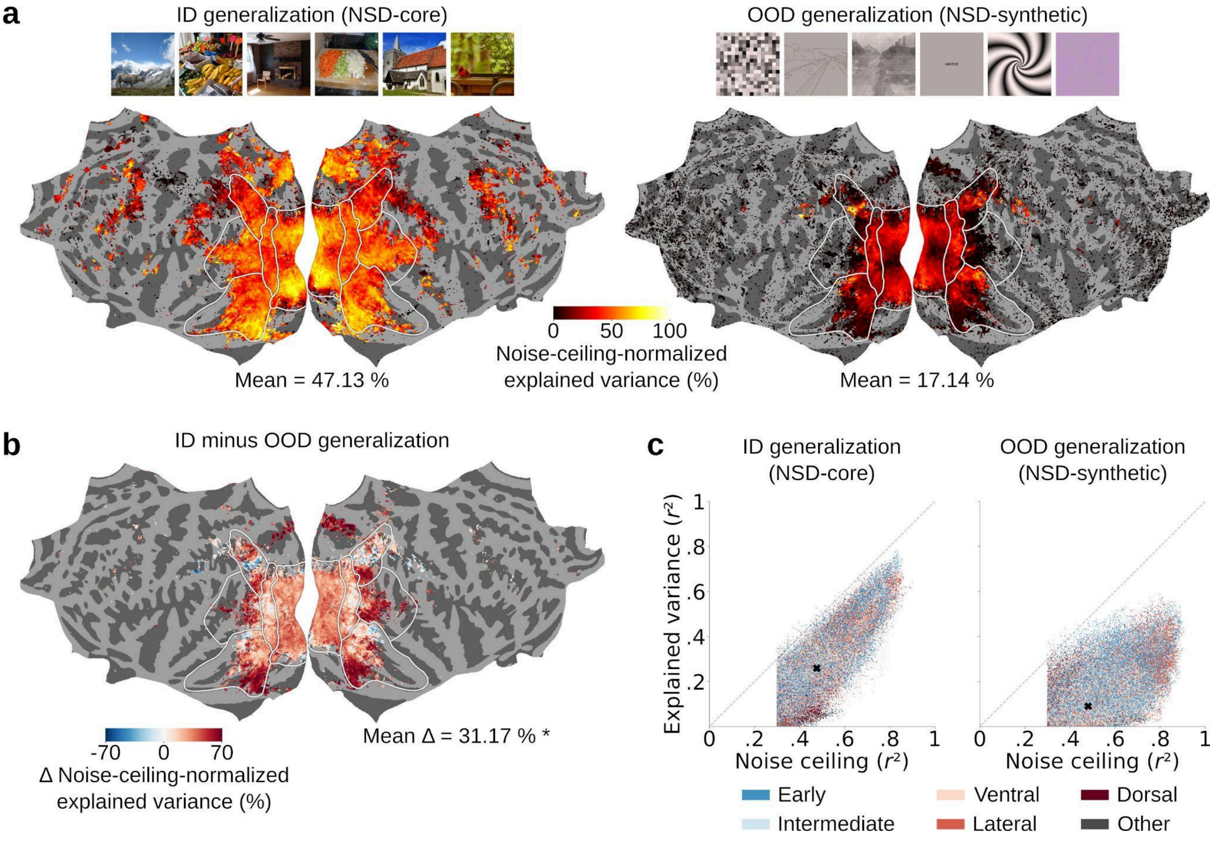

We built MoCo-based encoding models and tested them in-distribution (ID) on NSD-core as well as out-of-distribution (OOD) on NSD-synthetic. This figure shows results averaged across participants on flattened cortical surfaces. **a,** Model explained variance normalized by the noise ceiling. To reduce false positives, for each vertex we averaged results from only those participants with a noise ceiling greater than 0.3 (vertices for which no participant has a noise ceiling above 0.3 are not shown). **b,** Difference between ID and OOD noise-ceiling-normalized explained variance. For each vertex, we averaged results from only those participants with noise ceiling greater than 0.3 for both NSD-core and NSD-synthetic. The asterisk indicates a significant difference between the ID and OOD noise-ceiling-normalized explained variance scores ($P = 10^{-7}$, paired sample *t*-test, one-sided, $N = 8$ participants). **c,** Scatterplots of vertex-wise explained variance scores against their noise ceilings for ID (NSD-core) and OOD (NSD-synthetic) generalization tests, color coded by the visual stream that each vertex belongs to. For each vertex, we plotted results from only those participants with noise ceiling greater than 0.3 for both NSD-core and NSD-synthetic (same as in panel **b**). Black crosses indicate median scores across all vertices.

Supplementary Fig. 8 | In-distribution and out-of-distribution generalization differences between encoding models, controlling for session-specific effects

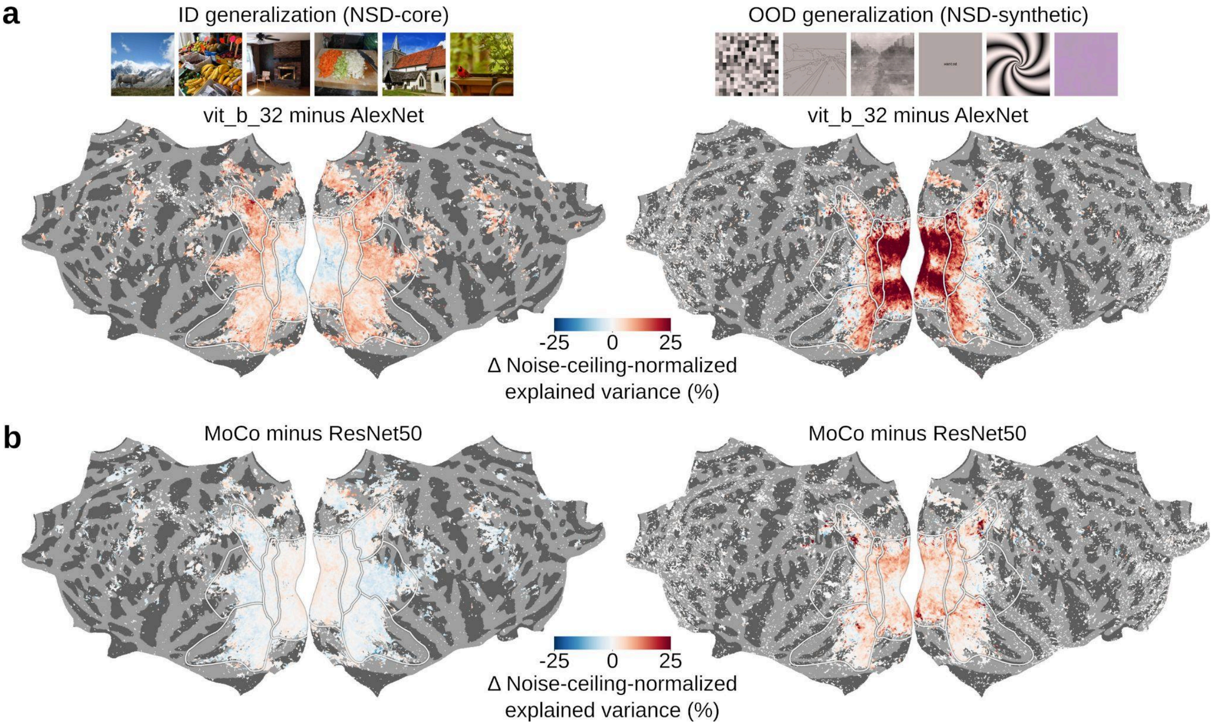

To control for session-specific effects, we trained and tested encoding models using fMRI responses from separate NSD-core scan sessions. For each participant, we trained the encoding models using image features and fMRI responses from all but the last 5 NSD-core scan sessions. We then tested these models' in distribution (ID) generalization on 284 image conditions that were only presented during NSD-core's last 5 scan sessions, and their out-of-distribution (OOD) generalization on the 284 image conditions from NSD-synthetic. This figure shows results averaged across participants on flattened cortical surfaces. To reduce false positives, for each vertex we averaged results from only those participants with a noise ceiling greater than 0.3 (vertices for which no participant has a noise ceiling above 0.3 are not shown). **a,** Difference between AlexNet and vit_b_32's noise-ceiling-normalized explained variance. **b,** Difference between MoCo and ResNet50's noise-ceiling-normalized explained variance.

Supplementary Fig. 9 | In-distribution generalization differences between encoding models based on individual MoCo and ResNet50 layers, trained with linear regression

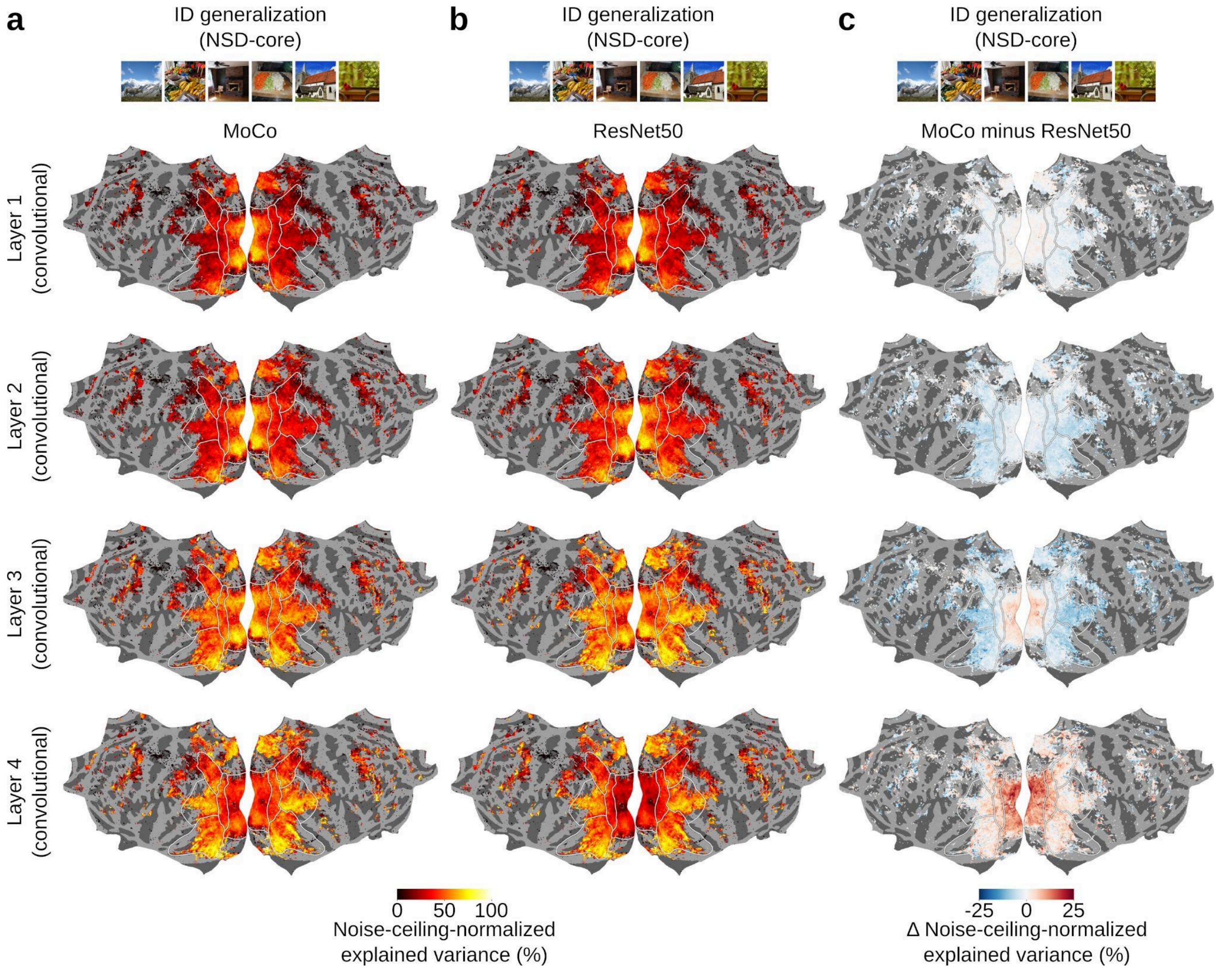

We trained separate encoding models for each of the 4 MoCo and ResNet50 layers: convolutional layer 1, convolutional layer 2, convolutional layer 3, and convolutional layer 4. The encoding models consisted of linear regressions that map each layer's image features (downsampled to 250 principal components using PCA) onto the fMRI responses of each vertex. We then tested these models in-distribution (ID) on NSD-core. This figure shows results averaged across participants on flattened cortical surfaces. To reduce false positives, for each vertex we averaged results from only those participants with a noise ceiling greater than 0.3 for NSD-core (vertices for which no participant has a noise ceiling above 0.3 are not shown). **a,** MoCo's ID explained variance normalized by the noise ceiling. **b,** ResNet50's ID explained variance normalized by the noise ceiling. **c,** Difference between MoCo and ResNet50's ID noise-ceiling-normalized explained variance.

Supplementary Fig. 10 | Out-of-distribution generalization differences between encoding models based on individual MoCo and ResNet50 layers, trained with linear regression

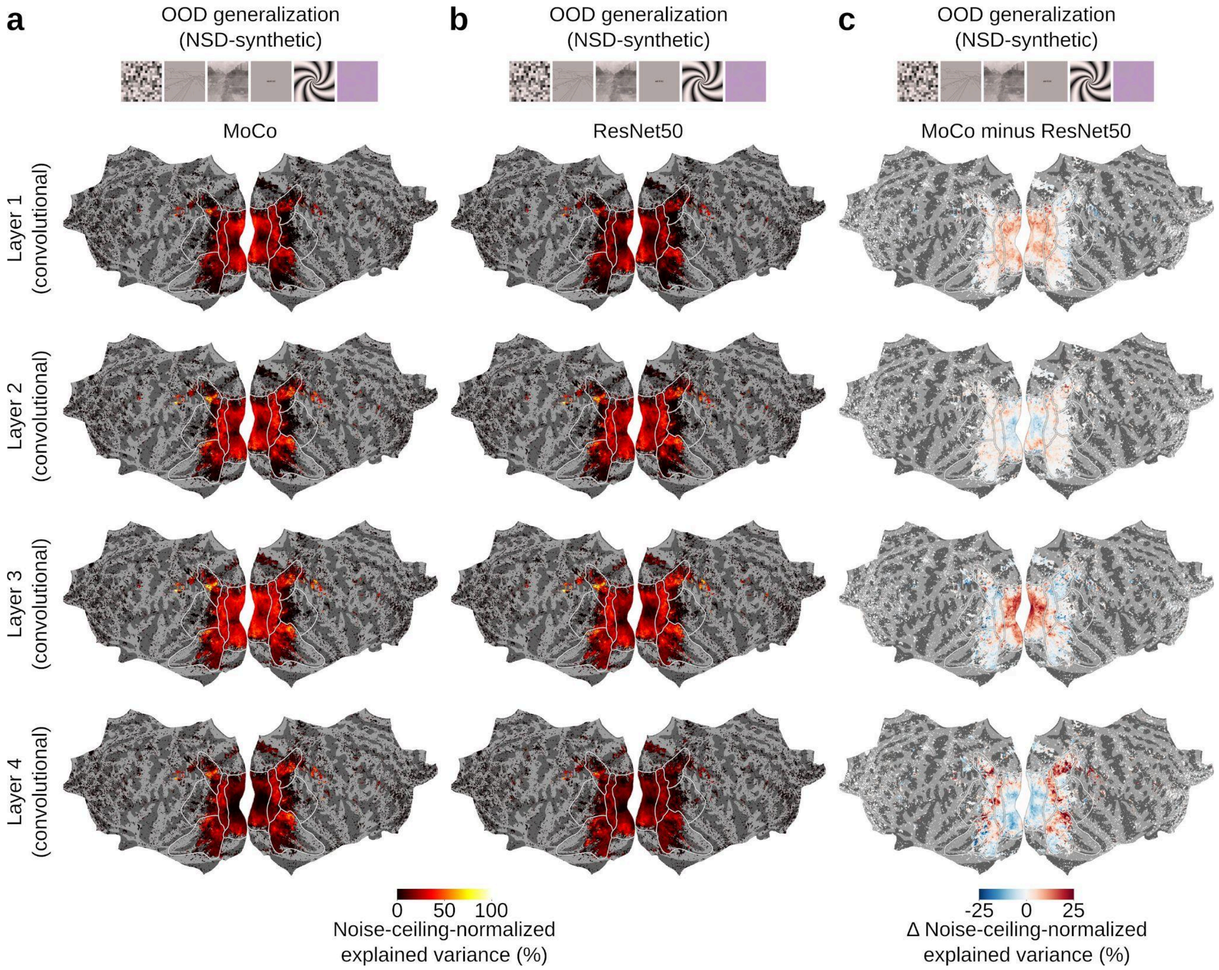

We trained separate encoding models for each of the 4 MoCo and ResNet50 layers: convolutional layer 1, convolutional layer 2, convolutional layer 3, and convolutional layer 4. The encoding models consisted of linear regressions that map each layer's image features (downsampled to 250 principal components using PCA) onto the fMRI responses of each vertex. We then tested these models out-of-distribution (OOD) on NSD-synthetic. This figure shows results averaged across participants on flattened cortical surfaces. To reduce false positives, for each vertex we averaged results from only those participants with a noise ceiling greater than 0.3 for NSD-synthetic (vertices for which no participant has a noise ceiling above 0.3 are not shown). **a,** MoCo's OOD explained variance normalized by the noise ceiling. **b,** ResNet50's OOD explained variance normalized by the noise ceiling. **c,** Difference between MoCo and ResNet50's OOD noise-ceiling-normalized explained variance.

Supplementary Fig. 11 | In-distribution generalization differences between encoding models based on individual MoCo and ResNet50 layers, trained with ridge regression

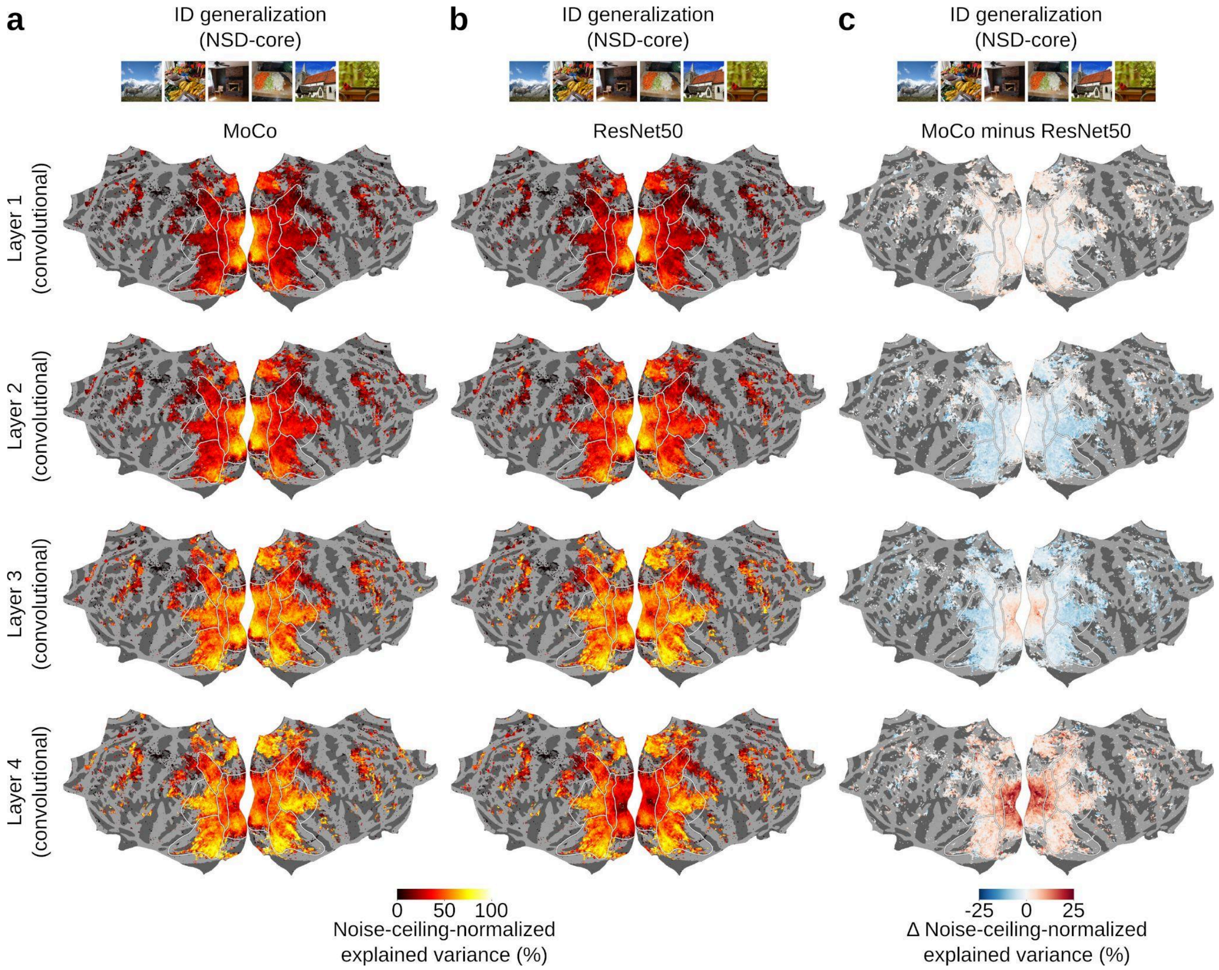

We trained separate encoding models for each of the 4 MoCo and ResNet50 layers: convolutional layer 1, convolutional layer 2, convolutional layer 3, and convolutional layer 4. The encoding models consisted of cross-validated ridge regressions that map each layer's image features onto the fMRI responses of each vertex. We then tested these models in-distribution (ID) on NSD-core. This figure shows results averaged across participants on flattened cortical surfaces. To reduce false positives, for each vertex we averaged results from only those participants with a noise ceiling greater than 0.3 for NSD-core (vertices for which no participant has a noise ceiling above 0.3 are not shown). **a,** MoCo's ID explained variance normalized by the noise ceiling. **b,** ResNet50's ID explained variance normalized by the noise ceiling. **c,** Difference between MoCo and ResNet50's ID noise-ceiling-normalized explained variance.

Supplementary Fig. 12 | Out-of-distribution generalization differences between encoding models based on individual MoCo and ResNet50 layers, trained with ridge regression

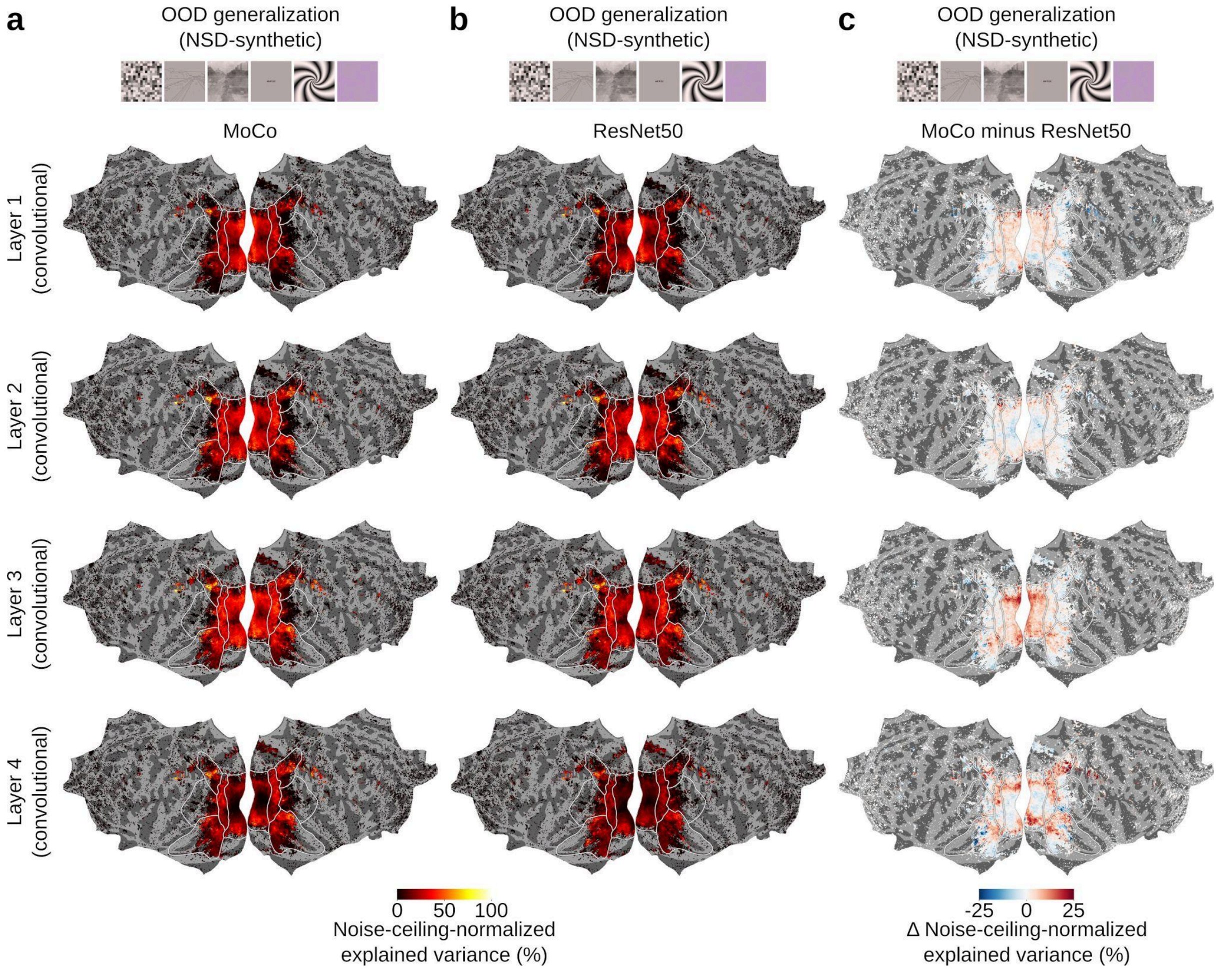

We trained separate encoding models for each of the 4 MoCo and ResNet50 layers: convolutional layer 1, convolutional layer 2, convolutional layer 3, and convolutional layer 4. The encoding models consisted of cross-validated ridge regressions that map each layer's image features onto the fMRI responses of each vertex. We then tested these models out-of-distribution (OOD) on NSD-synthetic. This figure shows results averaged across participants on flattened cortical surfaces. To reduce false positives, for each vertex we averaged results from only those participants with a noise ceiling greater than 0.3 for NSD-synthetic (vertices for which no participant has a noise ceiling above 0.3 are not shown). **a,** MoCo's OOD explained variance normalized by the noise ceiling. **b,** ResNet50's OOD explained variance normalized by the noise ceiling. **c,** Difference between MoCo and ResNet50's OOD noise-ceiling-normalized explained variance.

Supplementary Fig. 13 | Relationship between the degree of OOD and the generalization performance of AlexNet-based encoding models

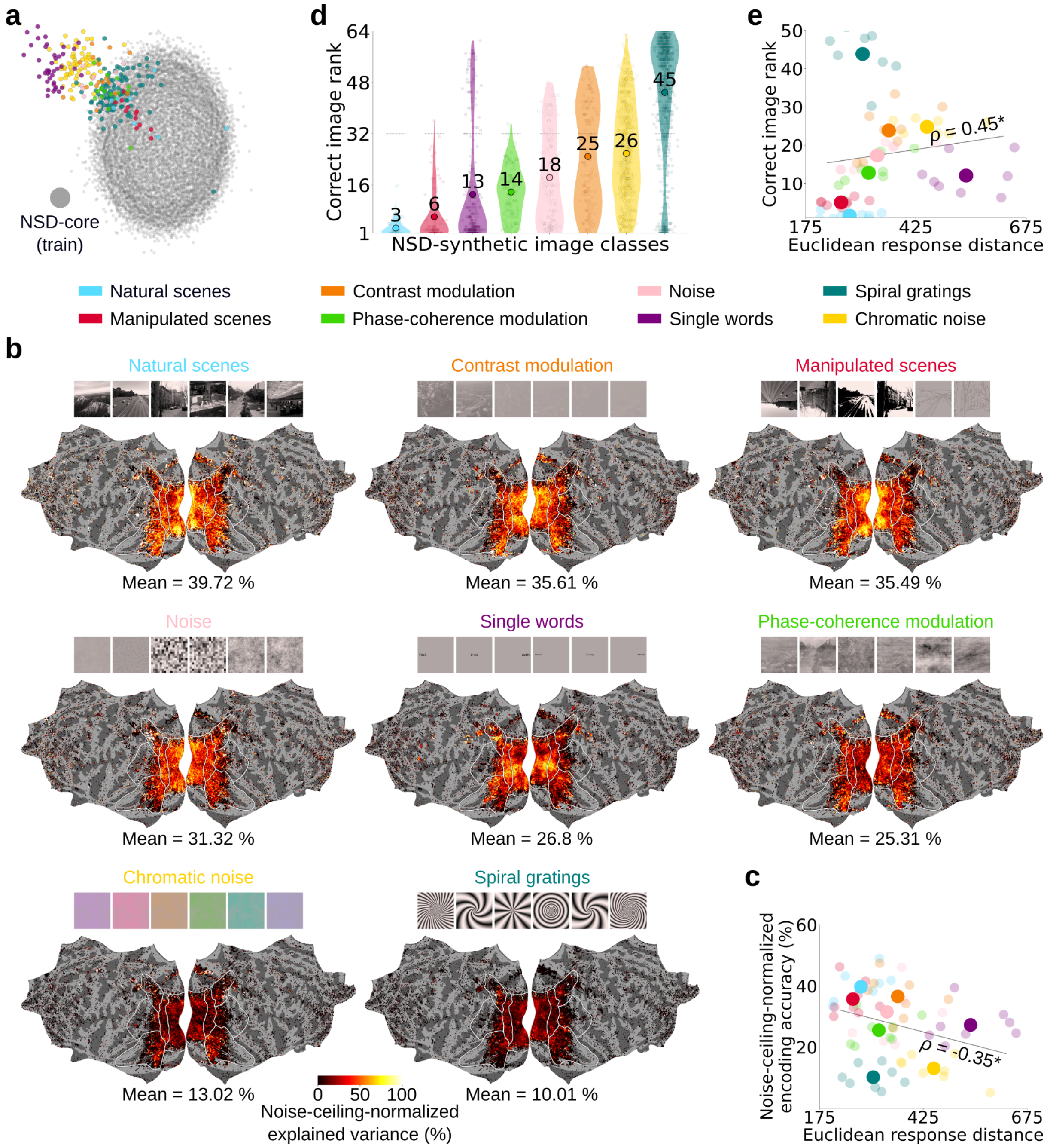

We trained AlexNet-based encoding models on NSD-core, and tested them independently for each NSD-synthetic image class. **a,** MDS embedding of trial-averaged fMRI responses (aggregated across participants) from NSD-core (gray dots) and from NSD-synthetic (colored dots, color-coded according to image class). **b,** Encoding models' explained variance for each NSD-synthetic image class, normalized by the noise ceiling. This figure shows results averaged across participants on flattened cortical surfaces. To reduce false positives, for each vertex we averaged results from only those participants with a noise ceiling greater than 0.3 (vertices for which no participant has a noise ceiling above 0.3 are not shown). **c,** Scatterplot indicating the relationship between the degree of OOD–that is, the degree of distributional distance–of fMRI responses and the encoding accuracy scores, across NSD-synthetic image classes. The x-axis represents the Euclidean distance in MDS embedding space

between fMRI responses for each NSD-synthetic image class and NSD-core (i.e., the distances in panel **a**). The y-axis represents the noise-ceiling normalized encoding accuracy of encoding models tested on each NSD-synthetic image class (i.e., the mean scores in panel **b**). **d,** Encoding models' zero-shot identification scores of the recorded fMRI response stimulus images, for each NSD-synthetic image class, on violin plots. The y-axis represents the correct image rank, that is, the position at which the correct image appears among the models' choices (choosing among 8 randomly selected images per NSD-synthetic image class, for a total of 64 images; the final identification scores reflect the average across 1,000 analysis iterations, each with a different random selection of 8 images per image class), with lower ranks indicating a more robust image identification. The colored violin shapes indicate the distribution of observations, with wider sections corresponding to higher density of values. Small transparent dots indicate the correct image rank for single images and participants. Large opaque dots indicate the average correct image rank across all participants and images within each NSD-synthetic image class, with the corresponding score reported above the dot. Horizontal dashed lines indicate chance identification scores. **e,** Scatterplot indicating the relationship between the degree of OOD of fMRI responses and the zero-shot image identification scores, across NSD-synthetic image classes. The x-axis represents the Euclidean distance in MDS embedding space between fMRI responses for each NSD-synthetic image class and NSD-core (i.e., the distances in panel **a**). The y-axis represents the average correct image rank across all images within each NSD-synthetic image class (i.e., the average scores in panel **d**). Large opaque and small transparent dots indicate participant-average and single participant scores, respectively. **c, e,** The asterisk indicates a significant correlation between response distance scores and encoding model performance ($P < 0.001$, one-sample t-test, one-sided, $N = 8$ participants).

Supplementary Fig. 14 | Relationship between the degree of OOD and the generalization performance of ResNet50-based encoding models

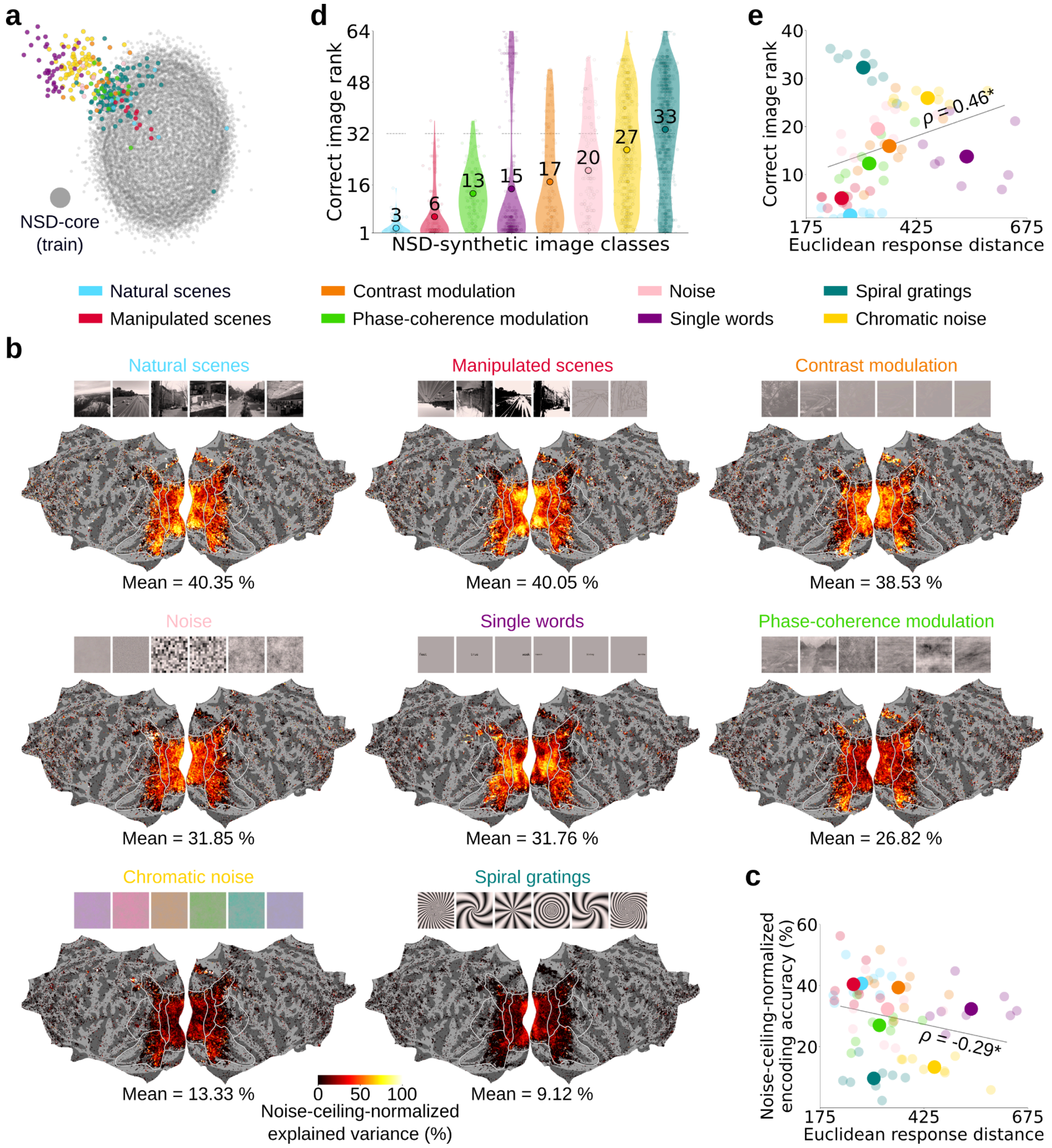

We trained ResNet50-based encoding models on NSD-core, and tested them independently for each NSD-synthetic image class. **a,** MDS embedding of trial-averaged fMRI responses (aggregated across participants) from NSD-core (gray dots) and from NSD-synthetic (colored dots, color-coded according to image class). **b,** Encoding models' explained variance for each NSD-synthetic image class, normalized by the noise ceiling. This figure shows results averaged across participants on flattened cortical surfaces. To reduce false positives, for each vertex we averaged results from only those participants with a noise ceiling greater than 0.3 (vertices for which no participant has a noise ceiling above 0.3 are not shown). **c,** Scatterplot indicating the relationship between the degree of OOD–that is, the degree of distributional distance–of fMRI responses and the encoding accuracy scores, across NSD-synthetic image classes. The x-axis represents the Euclidean distance in MDS embedding space

between fMRI responses for each NSD-synthetic image class and NSD-core (i.e., the distances in panel **a**). The y-axis represents the noise-ceiling normalized encoding accuracy of encoding models tested on each NSD-synthetic image class (i.e., the mean scores in panel **b**). **d,** Encoding models' zero-shot identification scores of the recorded fMRI response stimulus images, for each NSD-synthetic image class, on violin plots. The y-axis represents the correct image rank, that is, the position at which the correct image appears among the models' choices (choosing among 8 randomly selected images per NSD-synthetic image class, for a total of 64 images; the final identification scores reflect the average across 1,000 analysis iterations, each with a different random selection of 8 images per image class), with lower ranks indicating a more robust image identification. The colored violin shapes indicate the distribution of observations, with wider sections corresponding to higher density of values. Small transparent dots indicate the correct image rank for single images and participants. Large opaque dots indicate the average correct image rank across all participants and images within each NSD-synthetic image class, with the corresponding score reported above the dot. Horizontal dashed lines indicate chance identification scores. **e,** Scatterplot indicating the relationship between the degree of OOD of fMRI responses and the zero-shot image identification scores, across NSD-synthetic image classes. The x-axis represents the Euclidean distance in MDS embedding space between fMRI responses for each NSD-synthetic image class and NSD-core (i.e., the distances in panel **a**). The y-axis represents the average correct image rank across all images within each NSD-synthetic image class (i.e., the average scores in panel **d**). Large opaque and small transparent dots indicate participant-average and single participant scores, respectively. **c, e,** The asterisk indicates a significant correlation between response distance scores and encoding model performance ($P < 0.01$, one-sample t-test, one-sided, $N = 8$ participants).

Supplementary Fig. 15 | Relationship between the degree of OOD and the generalization performance of MoCo-based encoding models

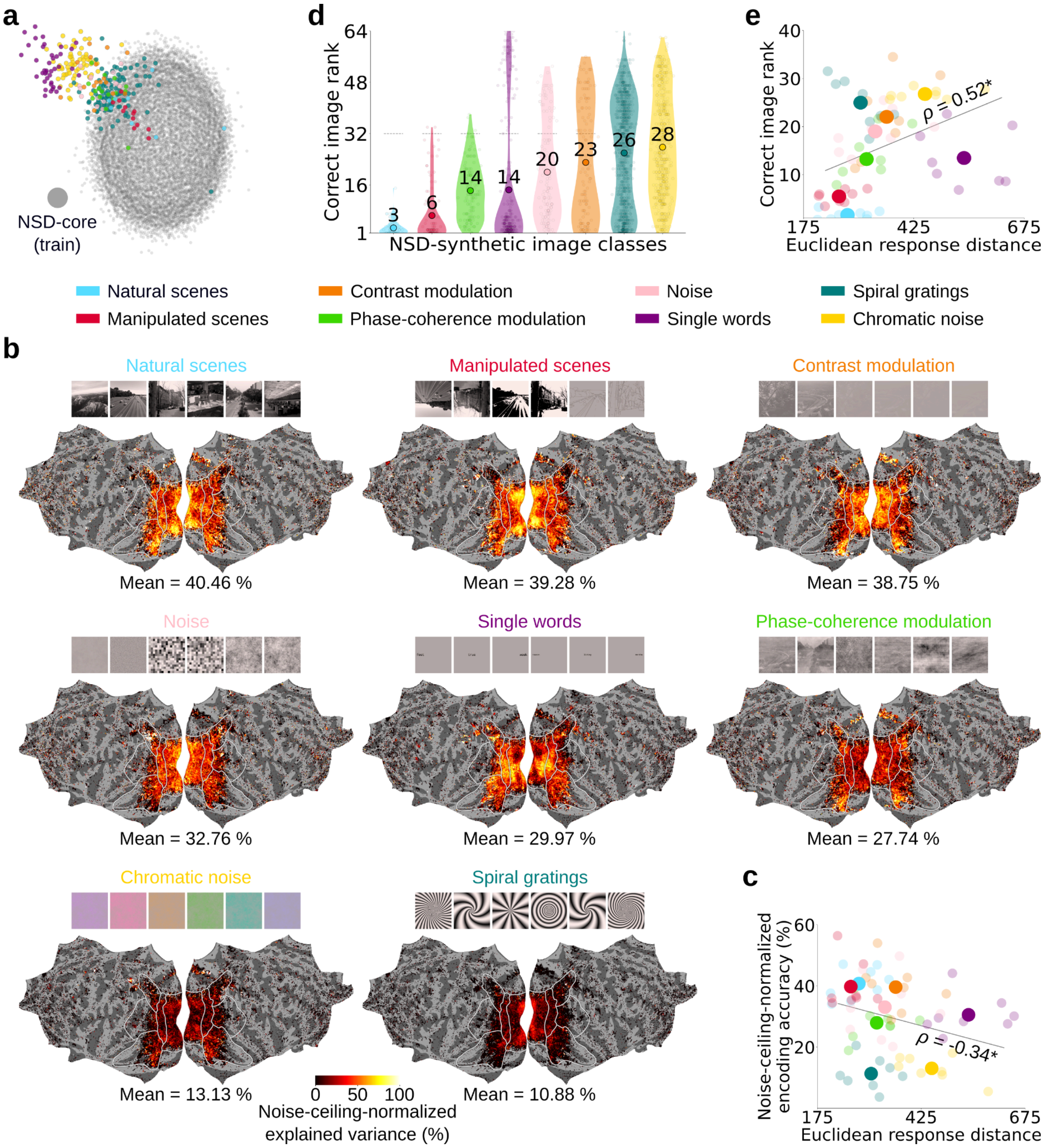

We trained MoCo-based encoding models on NSD-core, and tested them independently for each NSD-synthetic image class. **a,** MDS embedding of trial-averaged fMRI responses (aggregated across participants) from NSD-core (gray dots) and from NSD-synthetic (colored dots, color-coded according to image class). **b,** Encoding models' explained variance for each NSD-synthetic image class, normalized by the noise ceiling. This figure shows results averaged across participants on flattened cortical surfaces. To reduce false positives, for each vertex we averaged results from only those participants with a noise ceiling greater than 0.3 (vertices for which no participant has a noise ceiling above 0.3 are not shown). **c,** Scatterplot indicating the relationship between the degree of OOD–that is, the degree of distributional distance–of fMRI responses and the encoding accuracy scores, across NSD-synthetic image classes. The x-axis represents the Euclidean distance in MDS embedding space

between fMRI responses for each NSD-synthetic image class and NSD-core (i.e., the distances in panel **a**). The y-axis represents the noise-ceiling normalized encoding accuracy of encoding models tested on each NSD-synthetic image class (i.e., the mean scores in panel **b**). **d,** Encoding models' zero-shot identification scores of the recorded fMRI response stimulus images, for each NSD-synthetic image class, on violin plots. The y-axis represents the correct image rank, that is, the position at which the correct image appears among the models' choices (choosing among 8 randomly selected images per NSD-synthetic image class, for a total of 64 images; the final identification scores reflect the average across 1,000 analysis iterations, each with a different random selection of 8 images per image class), with lower ranks indicating a more robust image identification. The colored violin shapes indicate the distribution of observations, with wider sections corresponding to higher density of values. Small transparent dots indicate the correct image rank for single images and participants. Large opaque dots indicate the average correct image rank across all participants and images within each NSD-synthetic image class, with the corresponding score reported above the dot. Horizontal dashed lines indicate chance identification scores. **e,** Scatterplot indicating the relationship between the degree of OOD of fMRI responses and the zero-shot image identification scores, across NSD-synthetic image classes. The x-axis represents the Euclidean distance in MDS embedding space between fMRI responses for each NSD-synthetic image class and NSD-core (i.e., the distances in panel **a**). The y-axis represents the average correct image rank across and images within each NSD-synthetic image class (i.e., the average scores in panel **d**). Large opaque and small transparent dots indicate participant-average and single participant scores, respectively. **c, e,** The asterisk indicates a significant correlation between response distance scores and encoding model performance (P < 0.001, one-sample t-test, one-sided, N = 8 participants).

Supplementary Fig. 16 | Generalization performance of AlexNet-based encoding models across both naturalistic and synthetic stimulus images

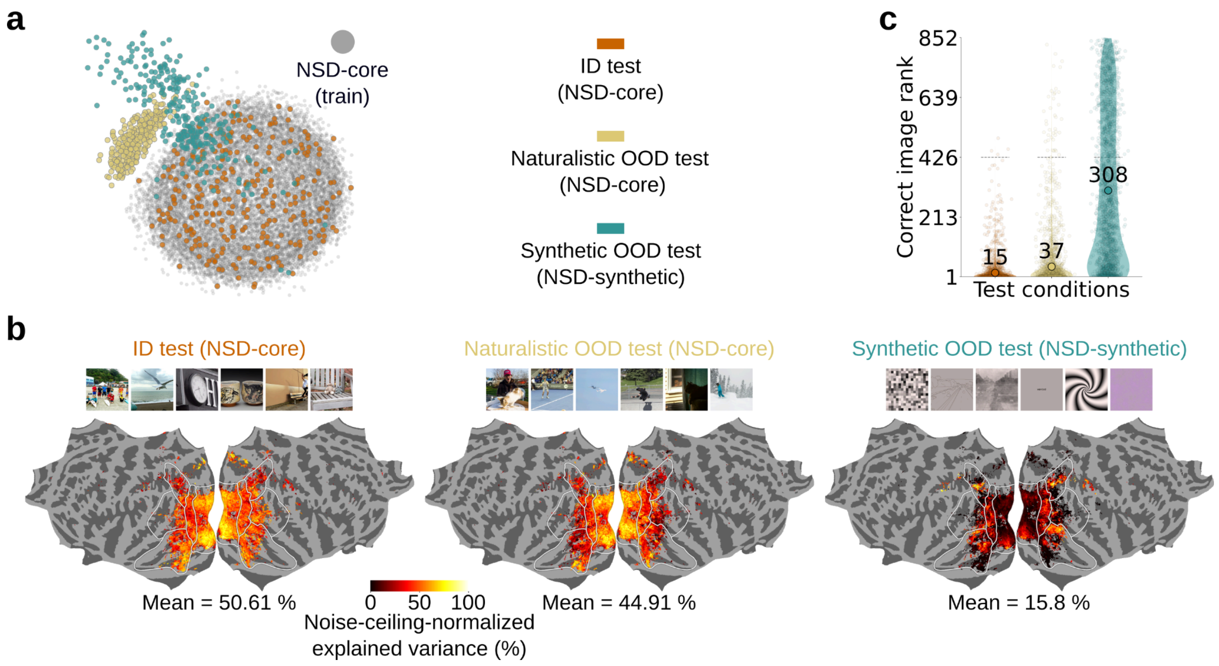

We trained AlexNet-based encoding models on NSD-core, and tested their generalization across three conditions: ID on NSD-core, naturalistic OOD on NSD-core, and synthetic OOD on NSD-synthetic. **a,** MDS embedding of trial-averaged fMRI responses (aggregated across participants) for the train images (gray dots), for the ID test images (orange dots), for the naturalistic OOD test images (yellow dots), and for the synthetic OOD test images (turquoise dots). **b,** Encoding models' explained variance for the three test conditions, normalized by the noise ceiling. This figure shows results averaged across participants on flattened cortical surfaces. To reduce false positives, for each vertex we averaged results from only those participants with a noise ceiling greater than 0.3 for both NSD-core and NSD-synthetic (vertices for which no participant has a noise ceiling above 0.3 are not shown). **c,** Encoding models' zero-shot identification scores of the recorded fMRI response stimulus images, for each of the three test conditions, on violin plots. The y-axis represents the correct image rank, that is, the position at which the correct image appears among the models' choices (choosing among all 852 images from the three test splits), with lower ranks indicating a more robust image identification. The colored violin shapes indicate the distribution of observations, with wider sections corresponding to higher density of values. Small transparent dots indicate the correct image rank for single images and participants. Large opaque dots indicate the average correct image rank across all participants and images within each test condition, with the corresponding score reported above the dot. Horizontal dashed lines indicate chance identification scores.

Supplementary Fig. 17 | Generalization performance of ResNet50-based encoding models across both naturalistic and synthetic stimulus images

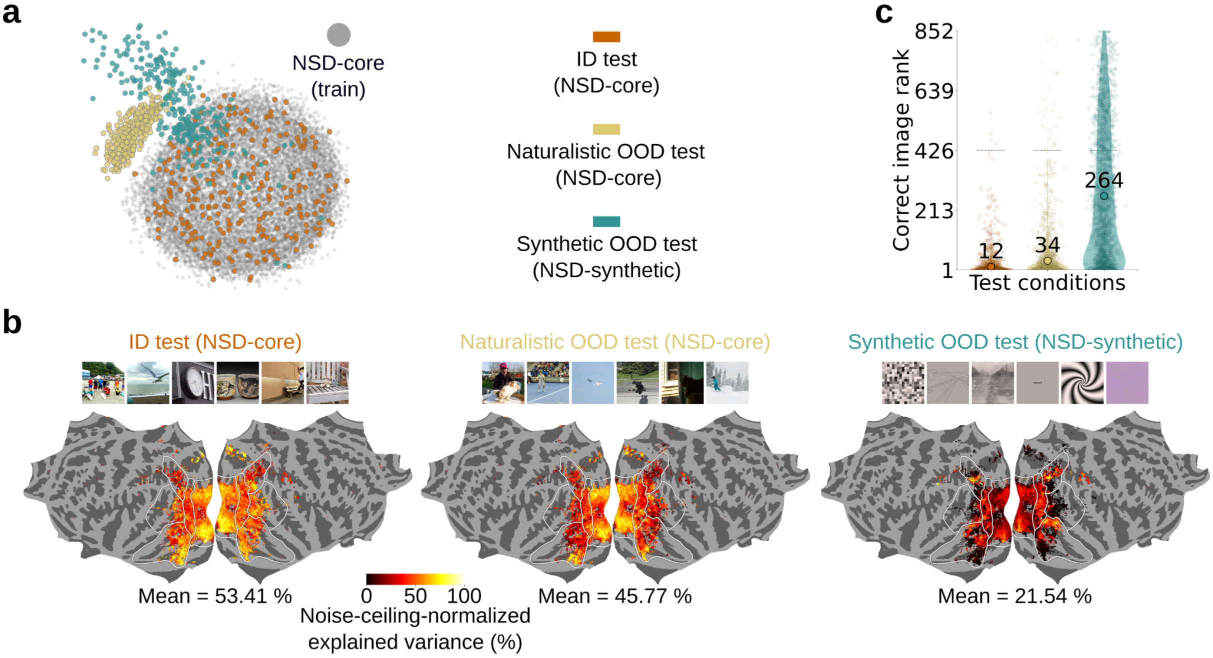

We trained ResNet50-based encoding models on NSD-core, and tested their generalization across three conditions: ID on NSD-core, naturalistic OOD on NSD-core, and synthetic OOD on NSD-synthetic. **a,** MDS embedding of trial-averaged fMRI responses (aggregated across participants) for the train images (gray dots), for the ID test images (orange dots), for the naturalistic OOD test images (yellow dots), and for the synthetic OOD test images (turquoise dots). **b,** Encoding models' explained variance for the three test conditions, normalized by the noise ceiling. This figure shows results averaged across participants on flattened cortical surfaces. To reduce false positives, for each vertex we averaged results from only those participants with a noise ceiling greater than 0.3 for both NSD-core and NSD-synthetic (vertices for which no participant has a noise ceiling above 0.3 are not shown). **c,** Encoding models' zero-shot identification scores of the recorded fMRI response stimulus images, for each of the three test conditions, on violin plots. The y-axis represents the correct image rank, that is, the position at which the correct image appears among the models' choices (choosing among all 852 images from the three test splits), with lower ranks indicating a more robust image identification. The colored violin shapes indicate the distribution of observations, with wider sections corresponding to higher density of values. Small transparent dots indicate the correct image rank for single images and participants. Large opaque dots indicate the average correct image rank across all participants and images within each test condition, with the corresponding score reported above the dot. Horizontal dashed lines indicate chance identification scores.

Supplementary Fig. 18 | Generalization performance of MoCo-based encoding models across both naturalistic and synthetic stimulus images

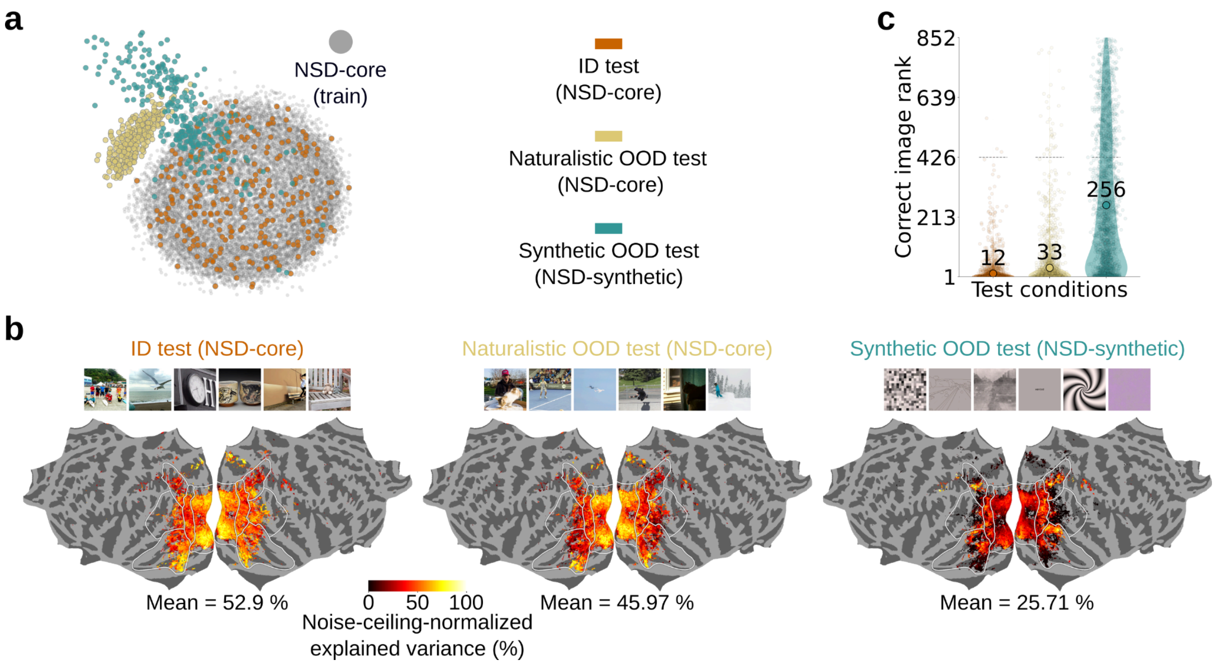

We trained MoCo-based encoding models on NSD-core, and tested their generalization across three conditions: ID on NSD-core, naturalistic OOD on NSD-core, and synthetic OOD on NSD-synthetic. **a,** MDS embedding of trial-averaged fMRI responses (aggregated across participants) for the train images (gray dots), for the ID test images (orange dots), for the naturalistic OOD test images (yellow dots), and for the synthetic OOD test images (turquoise dots). **b,** Encoding models' explained variance for the three test conditions, normalized by the noise ceiling. This figure shows results averaged across participants on flattened cortical surfaces. To reduce false positives, for each vertex we averaged results from only those participants with a noise ceiling greater than 0.3 for both NSD-core and NSD-synthetic (vertices for which no participant has a noise ceiling above 0.3 are not shown). **c,** Encoding models' zero-shot identification scores of the recorded fMRI response stimulus images, for each of the three test conditions, on violin plots. The y-axis represents the correct image rank, that is, the position at which the correct image appears among the models' choices (choosing among all 852 images from the three test splits), with lower ranks indicating a more robust image identification. The colored violin shapes indicate the distribution of observations, with wider sections corresponding to higher density of values. Small transparent dots indicate the correct image rank for single images and participants. Large opaque dots indicate the average correct image rank across all participants and images within each test condition, with the corresponding score reported above the dot. Horizontal dashed lines indicate chance identification scores.

Supplementary Fig. 19 | Generalization performance of encoding models to naturalistic in-distribution and out-of-distribution test splits chosen based on the image features of a deep neural network

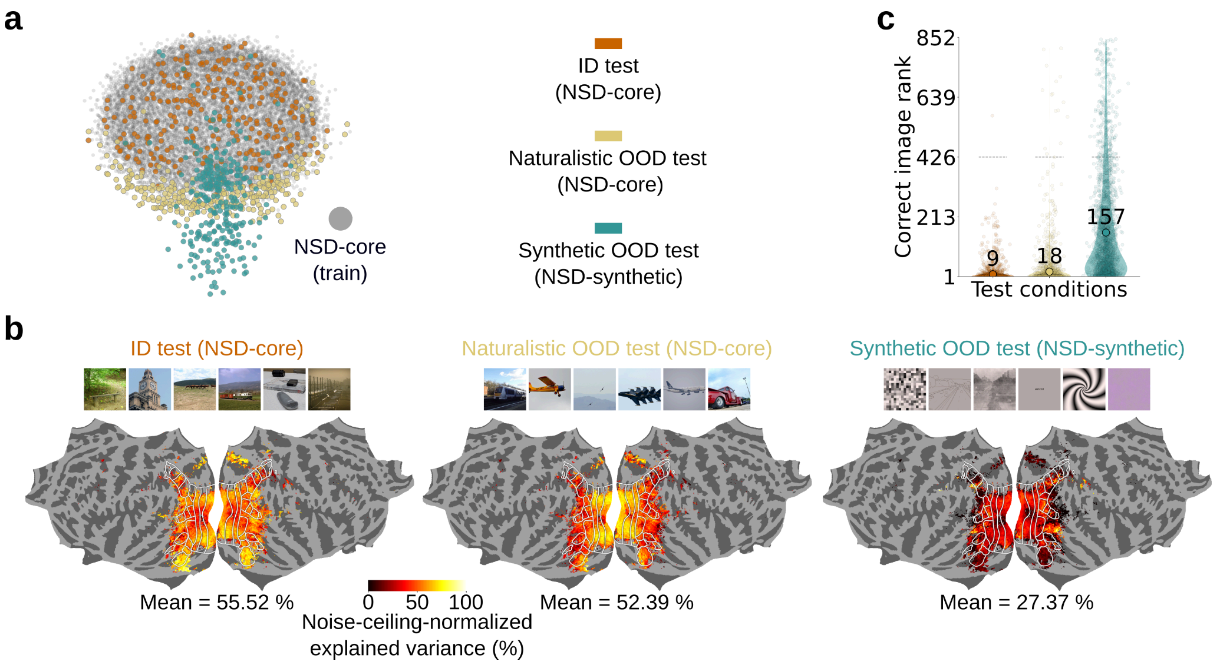

We trained vision-transformer-based encoding models on NSD-core, and tested their generalization across three conditions: ID on NSD-core, naturalistic OOD on NSD-core, and synthetic OOD on NSD-synthetic. To select the images of NSD-core's ID and naturalistic OOD test splits, we applied the MDS and clustering procedure described in the Results section of **Fig. 7** to the first 250 principal components of the image features of a vision transformer. **a,** MDS embedding of trial-averaged fMRI responses (aggregated across participants) for the train images (gray dots), for the ID test images (orange dots), for the naturalistic OOD test images (yellow dots), and for the synthetic OOD test images (turquoise dots). **b,** Encoding models' explained variance for the three test conditions, normalized by the noise ceiling. This figure shows results averaged across participants on flattened cortical surfaces. To reduce false positives, for each vertex we averaged results from only those participants with a noise ceiling greater than 0.3 for both NSD-core and NSD-synthetic (vertices for which no participant has a noise ceiling above 0.3 are not shown). **c,** Encoding models' zero-shot identification scores of the recorded fMRI response stimulus images, for each of the three test conditions, on violin plots. The y-axis represents the correct image rank, that is, the position at which the correct image appears among the models' choices (choosing among all 852 images from the three test splits), with lower ranks indicating a more robust image identification. The colored violin shapes indicate the distribution of observations, with wider sections corresponding to higher density of values. Small transparent dots indicate the correct image rank for single images and participants. Large opaque dots indicate the average correct image rank across all participants and images within each test condition, with the corresponding score reported above the dot. Horizontal dashed lines indicate chance identification scores.
